# Proteomic alterations underlie an association with teratozoospermia in obese mice sperm

**DOI:** 10.1186/s12958-019-0530-7

**Published:** 2019-10-24

**Authors:** Yuanhong Peng, Wenzhen Zhao, Fei Qu, Jia Jing, Yanqin Hu, Yue Liu, Zhide Ding

**Affiliations:** 1 Department of Histology, Embryology, Genetics and Developmental Biology, Shanghai Key Laboratory for Reproductive Medicine, Shanghai Jiao Tong University School of Medicine, Shanghai, 200025 China; 2 Department of Histology and Embryology, School of Basic Medical Science, Dali University, Dali, 671000 Yunnan China; 3 Institute of Reproductive Medicine, Dali University, Dali, 671000 Yunnan China

**Keywords:** Obesity, Teratozoospermia, Sperm proteome, CSPP1, CETN1

## Abstract

**Background:**

Obesity is a worldwide crisis impairing human health. In this condition, declines in sperm quality stem from reductions in sperm concentration, motility and increase in sperm deformity. The mechanism underlying these alterations remains largely unknown. This study, determined if obesity-associated proteomic expression patterns in mice sperm parallel those in spermatozoa obtained from obese humans.

**Methods:**

An obese mouse model was established via feeding a high-fat diet (HFD). Histological analysis identified testicular morphology and a computer assisted semen analyzer (CASA) evaluated sperm parameters. Proteome analysis was performed using a label-free quantitative LC-MS/MS system. Western blot, immunohistochemical and immunofluorescent analyses characterized protein expression levels and localization in testis, sperm and clinical samples.

**Results:**

Bodyweight gains on the HFD induced hepatic steatosis. Declines in sperm motility accompanied sperm deformity development. Differential proteomic analysis identified reduced cytoskeletal proteins, centrosome and spindle pole associated protein 1 (CSPP1) and Centrin 1 (CETN1), in sperm from obese mice. In normal weight mice, both CSPP1 and CETN1 were localized in the spermatocytes and spermatids. Their expression was appreciable in the post-acrosomal region parallel to the microtubule tracks of the manchette structure in spermatids, which affects spermatid head shaping and morphological maintenance. Moreover, CSPP1 was localized in the head–tail coupling apparatus of the mature sperm, while CETN1 expression was delimited to the post-acrosomal region within the sperm head. Importantly, sperm CSPP1 and CETN1 abundance in both the overweight and obese males decreased in comparison with that in normal weight men.

**Conclusion:**

These findings show that regionally distinct expression and localization of CETN1 and CSPP1 is strongly related to spermiogenesis and sperm morphology maintaining. Obesity is associated with declines in the CETN1 and CSPP1 abundance and compromise of both sperm morphology in mice and relevant clinical samples. This parallelism between altered protein expression in mice and humans suggests that these effects may contribute to poor sperm quality including increased deformity.

## Background

Obesity is a type of metabolic disease arising from an imbalance between caloric intake and metabolic expenditure. It can arise from a lack of physical exercise coupled with excessive food intake and genetic factors [1]. The worldwide size of the obese population has dramatically increased in recent years causing this disease to become a major problem impairing human health. Notably, in a 2018 report by the World Health Organization (WHO) (also in Global Health Observatory data repository), 1.9 billion overweight adults (BMI ≥ 25; age ≥ 18) were identified in 2016. In this population, male and female percentages were 39 and 40% respectively. Moreover, in this overweight population, there are over 650 million obese people (BMI ≥ 30) and the male and female percentages were 11 and 15% respectively. In general, obesity can increase the risk of developing hypertension, diabetes, respiratory diseases and cardiovascular diseases [2–7], as well as male infertility or subfertility. x

In the past decades, clinical studies showed that sperm quality decreases in the overweight and obese population, which is accompanied by decreases in both sperm concentration [8] and sperm motility [9, 10], abnormalities of acrosome reaction and sperm morphology, and increased sperm DNA damage [8, 11]. Several underlying pathophysiological mechanisms can link male obesity to poor sperm quality, including endocrine abnormalities, chronic inflammation and oxidative damage. For instance, male obesity can increase the estrogen and leptin levels, and decrease testosterone levels in the serum [12, 13]. Meanwhile, the pro-inflammatory cytokines (TNFα, IL-1, IL-6, etc.) in the serum, testis and seminal plasma are markedly up-regulated in obesity males [14]. The resulting chronic inflammation accompanied by oxidative stress in the male reproductive tract, directly impairs spermatogenesis in the testis and sperm maturation in the epididymis [15, 16].

On the other hand, in contrast to these established alterations in obese males, the negative impact of obesity on sperm quality is still poorly understood at the molecular level. The comparative proteomic approach is an informative tool to evaluate sperm functional characteristics. In our previous study, we used label-free quantitative LC-MS/MS proteomic analysis to contrast expression patterns in obesity-associated asthenozoospermic and normozoospermic individuals. We identified redox regulating chaperone ERp57 and actin binding protein ACTRT2 as two potential effectors of obesity-associated asthenozoospermia [17], but there is much inter-individual variation in the human population, due in large part to individual differences in lifestyle and genomic polymorphisms. As a result, it is difficult to elucidate some definite differences in sperm proteome related to obesity. Therefore, diet induced obesity animal models are widely employed to simulate human obesity caused by consuming a high fat diet. However, relatively few proteomic studies are available evaluating the contribution made by obesity to inducing declines in sperm quality.

We describe here the results of proteomic analysis to compare the differential effects of a high fat diet (HFD) and a control diet (CD) on sperm protein expression patterns. The results show that the declines in centrosome and spindle pole associated protein 1 (CSPP1) and Centrin 1 (CETN1) expression levels in mice fed a HFD may contribute to obesity-induced male subfertility.

## Methods

### Animals preparation and obese model establishment

All of the animal experiments were conducted following the International Guiding Principles for Biomedical Research Involving Animals, and the research program was approved by the Ethics Committee of Shanghai Jiao Tong University School of Medicine. The male 3-week-old C57BL/6 mice were purchased from the Shanghai Laboratory Animal Center, and housed in the Animal Center of Shanghai Jiao Tong University School of Medicine. After one-week of adaptation to a normal standardized diet, the mice were then randomly divided into two groups. One group was continuously fed for 10 weeks a high-fat diet (HFD) containing 23.3% casein, 0.3% L-cysteine, 8.5% corn starch, 11.7% maltodextrin, 20.1% sucrose, 5.8% cellulose, 2.9% soybean oil, 20.7% lard, 5.2% mineral mix, 1.2% vitamin mix, and 0.3% choline bitartrate. The other control group was fed for the same period with CD containing 19% casein, 0.2% L-cysteine, 29.9% corn starch, 3.3% maltodextrin, 33.2% sucrose, 4.7% cellulose, 2.4% soybean oil, 1.9% lard, 4.3% mineral mix, 0.9% vitamin mix, and 0.2% choline bitartrate. Both groups had ad libitum food and water access and were maintained on a 12 h light and 12 h darkness cycle. Body weight of every animal was recorded weekly. The mice fed with CD or HFD for 10 weeks were employed for the following experiments.

### Assessment of sperm parameters

The cauda epididymides separated from mice of each group were cut in pre-warmed (37 °C) Tyrode’s Buffer (Sigma-Aldrich, USA) and then placed into a 5% carbon dioxide incubator. After 15 min incubation, sperm motility, progressive motility and concentration were analyzed by computer-assisted sperm analysis (CASA) (Hamilton Thorne, USA). For teratozoospermia analysis, a sperm pellet was initially smeared on a glass slide. After reaching dryness at room temperature, the slide was fixed and stained as described in the Diff-Quick method (BRED Life Science Technology Inc., China). Then sperm morphology was observed under a microscope (Olympus, BX53, Japan) and the ratio of teratozoospermia was calculated in at least 200 sperm for each slide and repeated three times.

### Proteomic analysis

For proteomic analysis, sperm samples were collected from caudal epididymes by centrifugation in a 45% Percoll gradient (GE Healthcare, Waukesha, WI, USA) (800 g, 20 min, 4 °C) and then washed thrice with PBS. Six samples from the CD group and six samples from the HFD group were prepared for liquid chromatography tandem mass spectrometry (LC-MS) and performed as described [18]. All MS/MS spectra were searched using Proteome Discoverer 2.2 software against the mouse UniProt database and two missing cleavage sites were allowed. The tolerances of peptides and fragment ions were set at 6 ppm and 0.5 Da, respectively.

### Histological analysis

Tissues fixed in Bouin’s solution were embedded in paraffin, and specimens were sliced into 5 μm thick sections and mounted on glass slides, followed by deparaffinization and rehydration. The sectioned testicular and epididymal tissues were then stained with hematoxylin and eosin (H&E) and observed under a microscope (Olympus BX53).

### Western blot analysis

Western blot analyses were performed as modified as described before [18]. Testicular protein was separated by using 12% denaturing polyacrylamide gels after extracting and determining the concentration, then the protein was transferred to polyvinylidene difluoride (PVDF) membranes (Millipore, Germany). The membranes were blocked by using 5% bovine serum albumin (BSA) for 1 h, then incubated at 4 °C overnight with the primary antibodies against CSPP1 (Abcam, USA; 1:1000), CETN1 (Abcam, 1:2000), and β-actin (Abcam, 1:5000), followed by incubation with secondary antibody conjugated to HRP (Abgent, San Diego, CA, USA, 1:10000 dilution). Then enhanced chemiluminescence (Millipore, Germany) was employed to generate the signals detected by a Luminescent Image Analyzer (Image Quant LAS 4000, GE imagination at work, USA) according to the manufacturer’s protocol. Western blot was repeated at least three times to confirm the results reproducibility.

### Immunohistochemistry (IHC) analysis

IHC was performed using standard protocols. Paraffin sections were dewaxed and rehydrated, followed by antigen retrieval through boiling the tissue for 15 min in 10 mM citrate buffer, pH 6.0. Then the Histostain LAB-SA Detection kits (Invitrogen, MD, USA) were applied according to the manufacturer’s instructions. Primary antibody exposure against CSPP1 (1:100 dilution) or CETN1 (1:200 dilution) and the normal IgG (control) were performed overnight at 4 °C. The sections were stained using DAB and nuclei were counterstained with hematoxylin. Digital images were captured under a microscope (Olympus BX53).

### Immunofluorescence (IF) analysis

The sperm smears were prepared and then fixed with 4% paraformaldehyde for 20 min at 4 °C. The unspecific binding sites were blocked with 10% BSA/PBS for 60 min at room temperature, and the sperm samples were incubated with the primary antibodies against CSPP1 (1:200 dilution) or CETN1 (1:400 dilution) overnight at 4 °C. Then Alexa Fluor 555-labeled donkey anti–rabbit secondary antibodies (1:500 dilution, Jackson ImmunoResearch) were used. Nuclei were counterstained with DAPI (Sigma-Aldrich). The fluorescence signals were detected under a laser scanning confocal microscope (Carl Zeiss LSM-510, Germany).

### Semen samples preparation and immunoblot analysis

Human semen specimens, sperm parameters and relevant clinical data were obtained from Reproductive Medicine Center, Ruijin Hospital, Shanghai Jiao Tong University School of Medicine. Use of the semen samples was approved by the Ethics Committee of this institution and all experiments were performed in accordance with relevant guidelines and regulations. The clinical data of semen samples were divided into normal group (18 ≤ BMI < 25) and obese or overweight group (BMI ≥ 25) according to the human BMI, and then sperm motility and morphology were calculated. All the donors (22 to 38 years old), both normal and obese or overweight, gave written informed consent for the use of their leftover semen samples when all IVF treatments finished, and then their semen specimens were collected. Notably, individuals having a history of long-term medication, varicocoele, and infection as indicated by a high number of leukocytes in the semen were excluded from the study. Additionally, samples that were hyperviscous and necrozoospermia (sperm viability < 70%) were also excluded from the study.

Fresh human semen specimens were centrifuged (800 g, 10 min, 4 °C) and the sperm precipitates were dissociated in lysis buffer (6 M urea, 2 M thiourea, and 4% CHAPS). The separated sperm proteins were stored immediately at − 80 °C until further use. CSPP1 in sperm was detected using immunoblot analysis according to the protocol stated above.

### Statistical analysis

All data were analyzed by SPSS software (SPSS Statistic 23, Chicago, IL, USA), and the data are reported as mean ± STD. Comparisons between two groups were made using Student’s t-test appropriately. One-way analysis of variance (ANOVA) test was used assuming a two-tail hypothesis with *P* < 0.05. Differences were considered statistically different when *P* < 0.05.

## Results

**Fig. 1:**
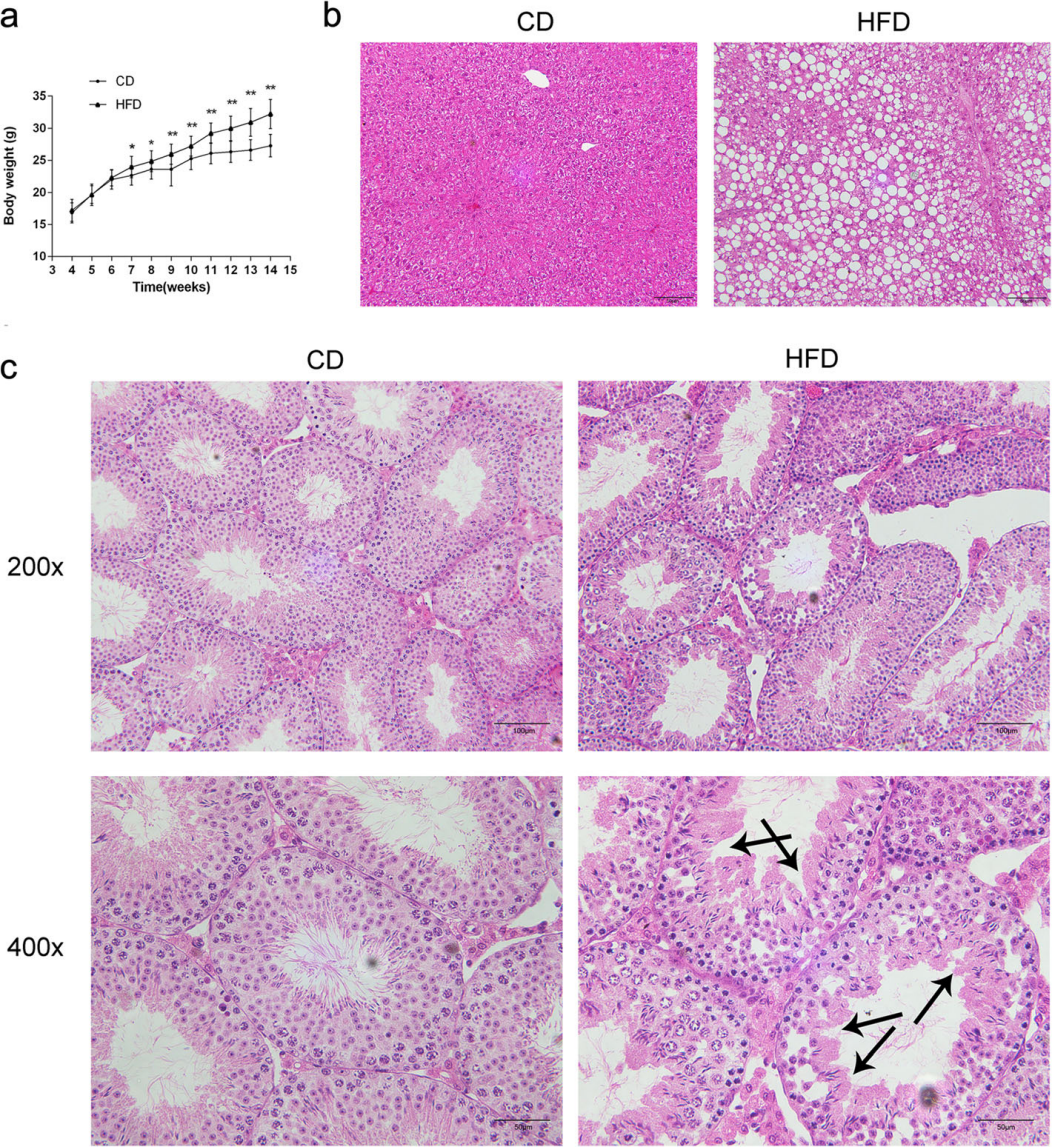
Body weight variations and hepatic and testicular morphological changes in mice fed a HFD. **a** Comparison of the body weights between the control diet (CD) group (*n* = 37) and high-fat diet (HFD) group (*n* = 37). Data are expressed as mean ± STD. *P < 0.05, **P < 0.01. **b** Hematoxylin & eosin-stained liver section morphology. Scale bars = 50 μm. **c** Hematoxylin & eosin staining compares testicular morphology in CD and HFD mice. Arrows indicate the poorly attached loose arrangement of spermatogenetic cells in the seminiferous epithelium of the HFD group. Scale bars = 50 or 100 μm

C57BL/6 mice fed a HFD for 4 weeks gained significantly more body weight than their age-matched littermates fed a CD. This difference in body weights between the two groups developed after four-weeks on the HFD. Subsequently, these body weight differences became more and more significant for an additional 7 weeks. As expected, mice on the HFD for 10 weeks were significantly heavier than age-matched littermates on the CD (32.25 ± 0.37 g vs. 27.30 ± 0.29 g, *n* = 37, *P* < 0.01) (Fig. 1a).

Histological analysis of the HFD group hepatic cells contained fat vacuoles in, which is indicative of the development of a serious hepatic steatosis and fatty liver (Fig. 1b).

### Alteration of sperm parameters and testicular morphology in obese mice

**Fig. 2:**
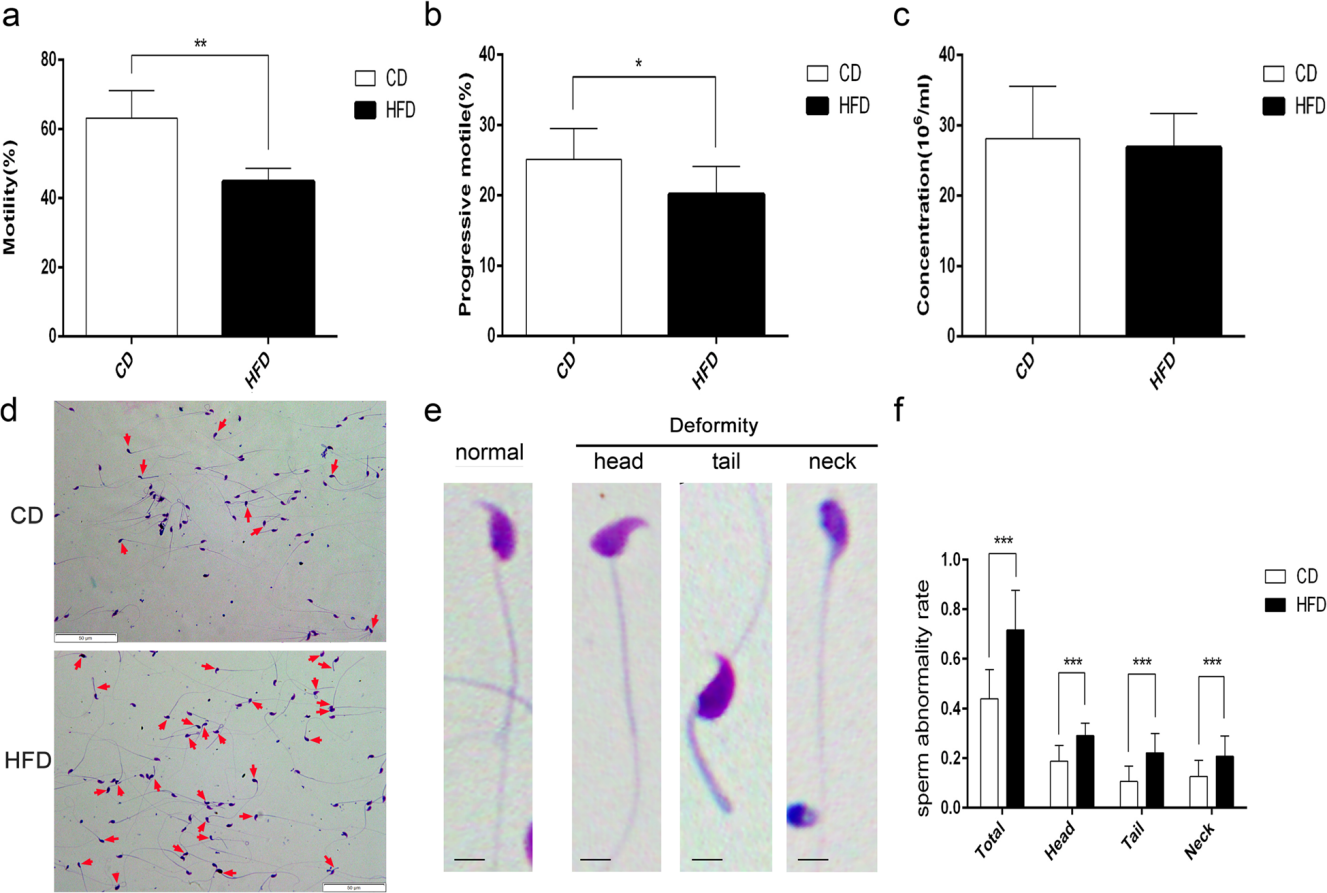
Changes in the sperm parameters in obese mice. **a-c** Sperm motility (**a**), progressive motility (**b**) and sperm concentration (**c**) analyzed by CASA. Data are expressed as mean ± STD. *P < 0.05, **P < 0.01. **d** Sperm morphology evaluated by Diff-Quick staining. Arrows indicate the abnormal sperm. Scale bars =50 μm. **e** The enlarged images exhibit normal sperm in the CD group and prominent deformity in sperm head, neck and tail in the HFD group. Scale bars =10 μm. **f** Deformed sperm ratios calculated from eight independent experiments and measured 200 spermatozoa at least in each independent experiment. Data are expressed as mean ± STD. *P < 0.05, **P < 0.01

CASA evaluation of the sperm parameters revealed that the HFD group sperm motility (44.80 ± 1.21, *n* = 10) and progressive motility (20.10 ± 1.27, *n* = 10) were significantly decreased in comparison to those of the CD group (percentage of sperm motility: 63.10 ± 2.83, *n* = 10, *P* < 0.01. Figure 2a; percentage of progressive motility: 25.10 ± 1.40, *n* = 10, *P* < 0.05 Fig. 2b). Moreover, the HFD group teratozoospermia ratio (total sperm deformity: 71.59 ± 16.03%, sperm head deformity: 29.13 ± 5.10%, sperm tail deformity: 22.25 ± 7.80%, sperm neck deformity: 20.83 ± 8.14%; *n* = 15) was significantly larger than that of the CD group (total sperm deformity: 44.04 ± 11.69%, sperm head deformity: 18.82 ± 6.41%, sperm tail deformity: 10.67 ± 6.25%, sperm neck deformity: 12.60 ± 6.54%; *n* = 15, *P* < 0.05 Fig. 2d-f). However, there was no difference in sperm concentration between the HFD and CD groups (26.81 ± 1.54 million per ml vs. 28.09 ± 2.37 million per ml, *n* = 10, *P* > 0.05 Fig. 2c).

Moreover, testicular morphological analysis showed that the HFD group morphology was disrupted compared with that in the CD group. The HFD group testicular sections had loose adhesions between the spermatogenetic cells and Sertoli cells, which disrupted spermatogenetic cell attachments and their organization (Fig. 1c).

### Differential sperm protein expression patterns between HFD group and CD group

**Fig. 3:**
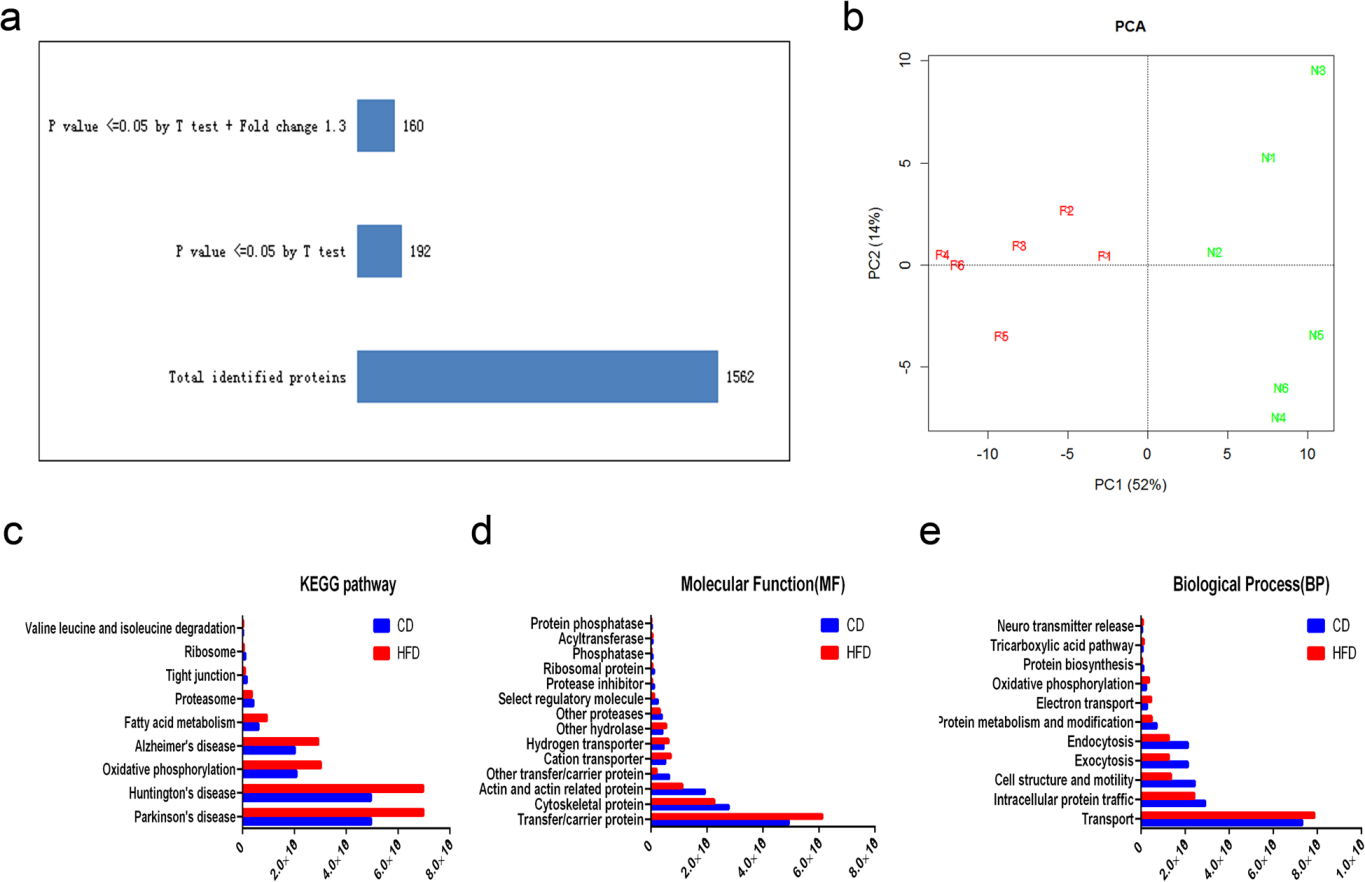
Quantitative proteomic profiling of sperm. **a** Proteomic analysis of total and differentially expressed proteins. **b** Principal component analysis of sperm protein in CD and HFD groups according to their protein profile data. **c-e** Functional categories of differentially expressed proteins. Major subgroups of molecular functions (**c**), biological processes (**d**) and KEGG pathways (**e**) identified in the sperm proteome of CD and HFD groups. For more information, see Additional file 1: Table S1

LC − MS/MS in conjunction with Maxquant analysis of 6 sperm samples selected from the CD and HFD groups provided quantitative proteomic profiles of these two groups. One thousand five hundred and sixty-two sperm proteins were identified in total (Fig. 3a), and then two samples were chosen randomly and the reproducibility of the MS analysis was determined. The results indicate that there is an excellent correction between the two groups (R^2^ = 0.997). The abundance of 192 proteins was significantly different between the CD and HFD groups (Fig. 3a, Additional file 1: Table S1). Principal component analysis (PCA) of the protein expression contents showed that all samples in each group had similar protein expression profiles, whereas those taken from different groups were clearly different from each other (Fig. 3b). Kyoto Encyclopedia of Genes and Genomes (KEGG) databases were employed to search for functional annotation terms (FATs) and pathways which are enriched in the proteins whose abundance is dissimilar between the two groups. The KEGG analysis revealed that most of the differentially expressed proteins were related to oxidative phosphorylation, Parkinson’s disease and Alzheimer’s disease, while the others were relevant to Huntington’s disease, tight junction, fatty acid metabolism, components of proteasome mediated valine, leucine and isoleucine degradation (Fig. 3c). Gene ontology (GO) analysis of the differentially expressed proteomes showed that their functional classifications were similar to the aforementioned parameters (Fig. 3d-e).

Among these differentially expressed proteins, some are related to cytoskeleton, such as calicin [19], cylicin [19, 20], myosin [21], dynein [22, 23] and septin [24], which are thought to participate in spermiogenesis and sperm motility. More importantly, the proteomic analysis data also showed that the sperm CETN1 and CSPP1 expression levels decreased in the HFD group. CENT1 is a calmodulin-like Ca^2+^ binding protein which participates in spermiogenesis [25]. On the other hand, little is known about CSPP1 expression and function and its role in spermatogenesis or sperm function is unclear. These declines in CETN1 and CSPP1 expression patterns prompted us to determine if they affect sperm fertility.

### Expression of CSPP1 and CETN1 in mice testis and sperm

Western blot analyses showed that CSPP1 and CETN1 are widely expressed in many tissues including liver, spleen, lung, kidney, brain, testis and ovaries. The results shown in Fig. 5a indicate that their expressions are enriched in testis relative to those reported in the aforementioned tissues. Their testicular abundance suggested that they have important roles in this tissue.

**Fig. 4:**
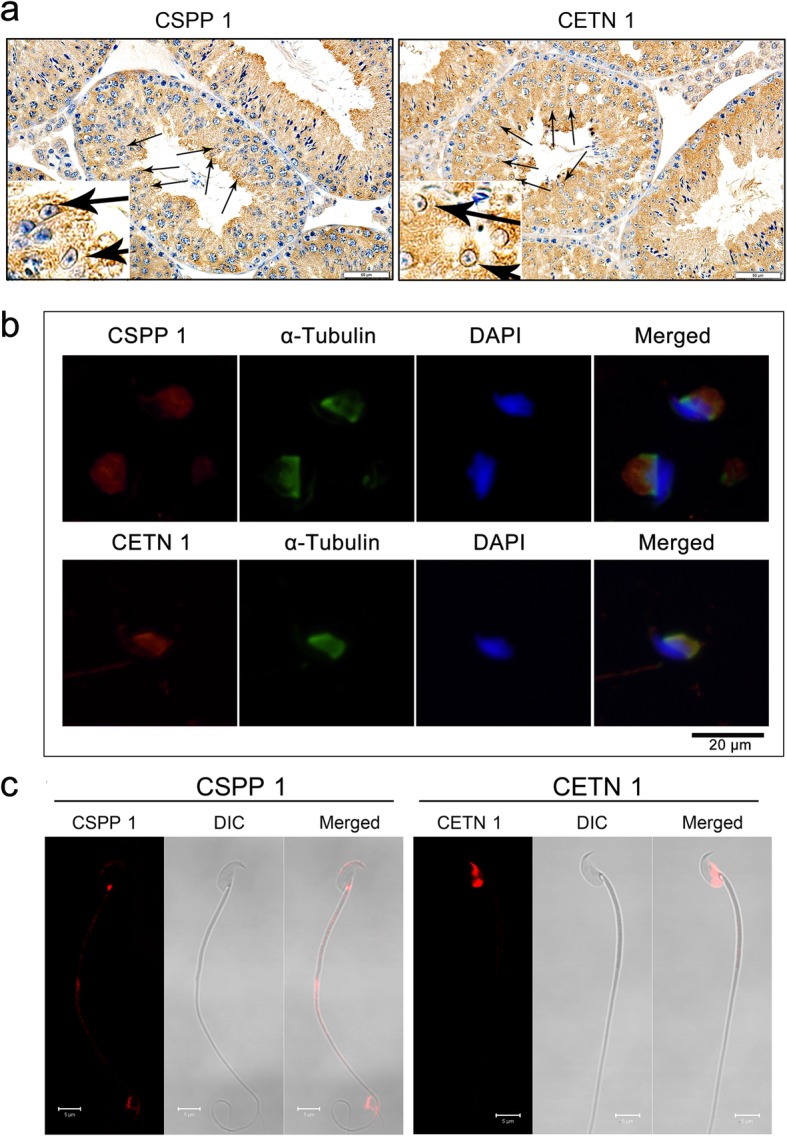
Localization of CSPP1 and CETN1 in normal mice testis and sperm. **a** Localization of CSPP1 and CETN1 in mice testis evaluated by immunohistochemistry staining. Arrows indicate the distribution of CSPP1 and CETN1 in spermatids. Scale bars = 50 μm. **b** Immunofluorescent analysis shows that CSPP1 and CETN1 co-localized with α-tubulin (indicating manchette structure) in spermatids, respectively. Scale bars = 20 μm. **c** Localization of CSPP1 and CETN1 in mice sperm. Scale bars = 5 μm

IHC analysis revealed that both CSPP1 and CETN1 are visibly expressed in spermatocytes and spermatids in the seminiferous epithelium, especially around the distal half of the nucleus in the spermatids (Fig. 4a). It is known that this region consists of microtubules having an enriched manchette structure that is responsible for spermatid shaping and maintenance of sperm head morphology. IF staining specifically showed that both CSPP1 and CETN1 co-localized with α-tubulin in the post-acrosomal region of the spermatids (Fig. 4b), suggesting their potential roles in sperm head remodeling during spermiogenesis.

Moreover, IF sperm analysis revealed that CSPP1 localized intensely in the head–tail coupling apparatus of mature sperm, and CETN1 localized in the post-acrosomal region of the sperm head (Fig. 4c). All of these results suggested that these two proteins may play some important roles in spermiogenesis and maintenance of sperm morphology.

### HFD-induced suppression of CSPP1 and CETN1 expression

**Fig. 5:**
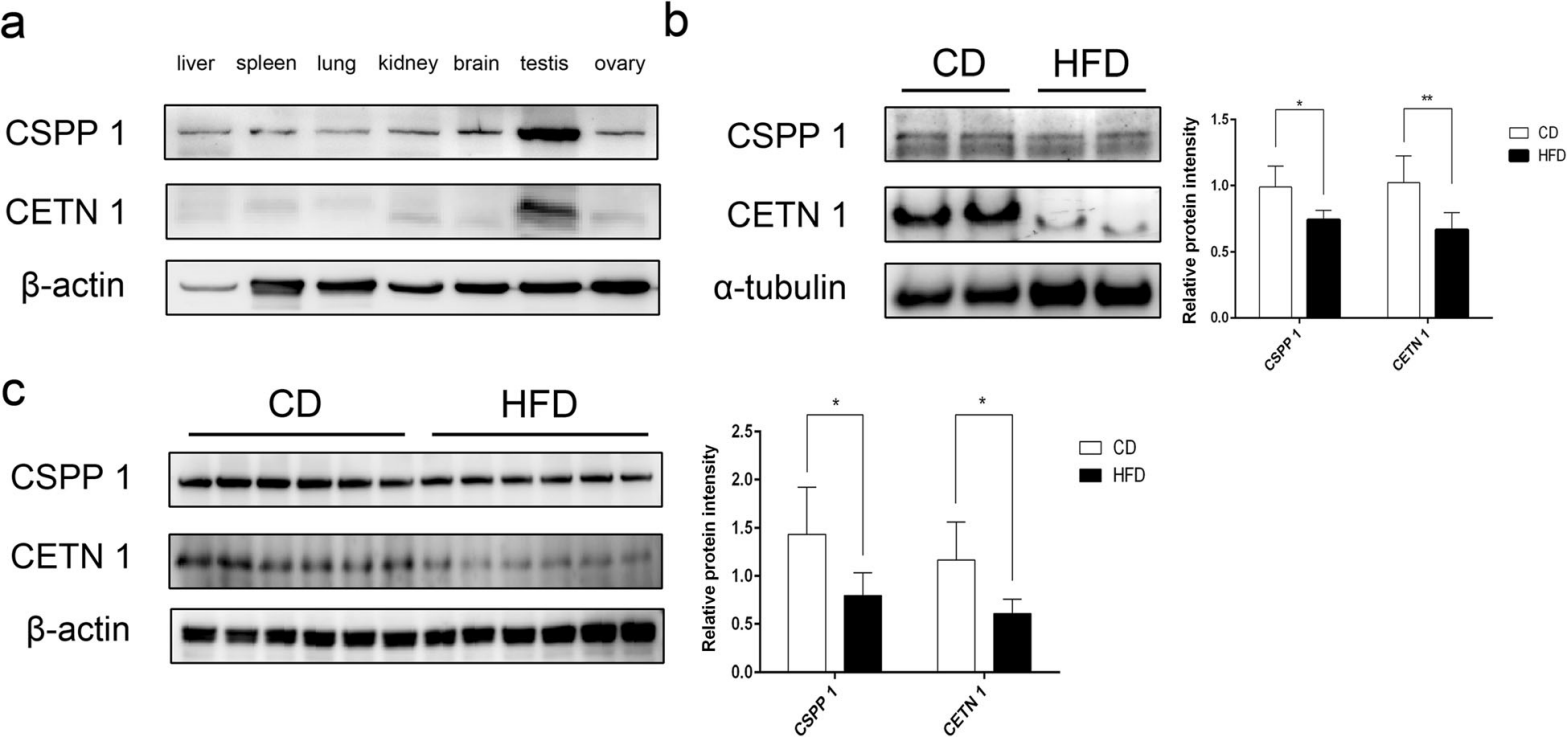
Comparison of CSPP1 and CETN1 protein expression levels in varied mouse organs and in obese mice testis and sperm. **a** Western blot analysis of CSPP1 and CETN1 in liver, spleen, lung, kidney, brain, testis and ovary of normal mice. **b** Western blot analysis of CSPP1 and CETN1 in sperm from CD and HFD groups. Densitometric analysis evaluated relative protein levels and were normalized expressed as ratios of target proteins and α-tubulin in twelve independent samples, respectively. **c** Western blot analysis of CSPP1 and CETN1 in testes from CD and HFD groups. Densitometric analysis determined relative protein levels which were calculated as a ratio between target proteins and β-actin from six independent samples respectively. Data were expressed as mean ± STD. *P < 0.05, **P < 0.01

Western blot analyses confirmed that the HFD reduced the sperm CSPP1 and CETN1 expression levels below those in the CD group (Fig. 5b, c). Combined with their function in regulating the cytoskeletal architecture, we presumed that reduced testicular CSPP1 and CETN1 expressions levels in the HFD group contributes to their high ratio of sperm deformity.

### Relationship between CSPP1 expression and sperm quality in clinical samples

**Fig. 6:**
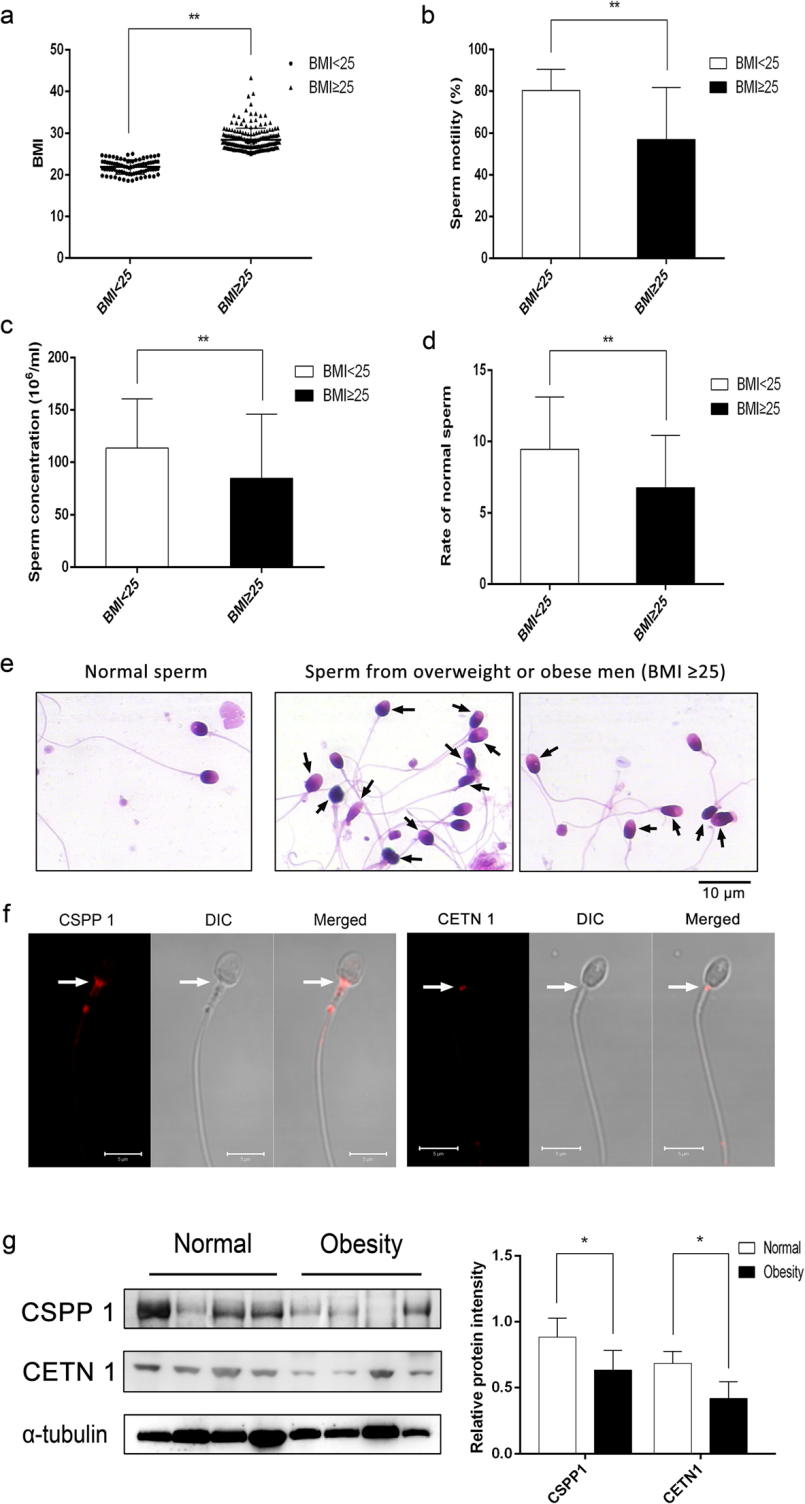
Diagnostic sperm parameters of normal and overweight or obese individuals and reduced abundance of CSPP1 and CETN1 in sperm from these individuals. **a-d** Body mass index (BMI) (**a**), sperm motility (**b**), sperm concentration (**c**) and sperm deformity (**d**) were calculated in normal (BMI < 25, *n* = 82) and overweight or obese (BMI ≥ 25, *n* = 190) individuals respectively. Data are expressed as mean ± STD. **P < 0.01. **e** Sperm morphology evaluated by Diff-Quick staining. Arrows indicate the abnormal sperm. Scale bars =10 μm. **f** CSPP1 and CETN1 localization in human sperm evaluated by immunofluorescent staining. Arrows indicated the positive signals in spermatozoa. Scale bars = 5 μm. **g** Western blot analysis of CSPP1 and CETN1 in human sperm from normal and overweight or obese individuals, respectively. Densitometric analysis evaluated the relative protein levels by a ratio of CSPP1 or CETN1 to α-tubulin in eight independent samples, respectively. Data are expressed as mean ± STD. *P < 0.05

Even though the function of CENT1 is known in spermatogenesis, there is scant information regarding the role of CSPP1 and CETN1 in obesity associated teratozoospermia. To determine if CSPP1 and CETN1 expressions correlate with sperm deformity, CSPP1 expression levels were compared with sperm quality in clinical samples obtained from normal males (BMI = 21.88 ± 1.72, *n* = 82) and overweight or obese males (BMI = 28.43 ± 2.82, *n* = 190, *P* < 0.01). The age of normal males (31.78 ± 5.79, *n* = 82) and overweight or obese males (32.73 ± 4.91, *n* = 190) was similar to each other (*P* = 0.273). The results shown in Fig. 6a compare the sperm motility and concentration of controls and overweight or obese individuals. In the overweight and obese group, sperm motility was 56.93 ± 24.80% and sperm concentration was 84.84 ± 61.08 (*n* = 190) whose values were both significantly less than those in the controls (sperm motility: 80.25 ± 10.13, sperm concentration: 113.35 ± 47.19, *n* = 82, *P* < 0.01) (Fig. 6b, c). Meanwhile, the ratio of sperm with normal morphology in overweight or obesity males (6.77 ± 3.66%, *n* = 190) is significantly less than in that in normal males (9.45 ± 3.66%, *n* = 82, *P* < 0.01) (Fig. 6d, e).

Moreover, IF analysis showed that CSPP1 and CETN1 were also localized in the head–tail coupling apparatus of human sperm (Fig. 6f), which coincides with their centrosome localization and flagellar outgrowth ability [25–27]. Additionally, immunoblot analysis showed that CSPP1 and CETN1 contents were significantly reduced in sperm from overweight or obesity males compared with that from normal males, which is in agreement with the results in the mice of HFD group (Fig. 6g). These results strongly suggest that declines in CSPP1 and CETN1 expression levels in sperm of the HFD group and clinical samples of obese males are associated with sperm deformity or teratozoospermia.

## Discussion

As obesity prevalence is continuously increasing worldwide, there is accumulating evidence indicating a correlation between obesity and reduced male fertility. The results of many clinical investigations reveal that there is a possible association between obesity and the occurrence of abnormal sperm parameters. They include decreases in sperm concentration, motility and increased incidence of deformities [28, 29]. However, the underlying mechanisms are still poorly understood regarding how obesity leads to declines in sperm quality and fertility. In our previous report, the HFD mice developed a remarkable impairment of sperm function, including reduced sperm motility and abnormal sperm morphology [16]. Herein, we describe the differences in the spermatozoa proteomes between the HFD and CD groups. The results clearly show that adequate expression levels of CSPP1, a cytoskeletal related protein, is potentially related to the maintenance of normal sperm morphology.

Sperm quality is affected by pathophysiological sequelae that include altered hormone levels, chronic inflammation and generation of excessive reactive oxygen species in the reproductive system. Moreover, there is an increasing awareness that male obesity increases the risk of oligozoospermia, asthenozoospermia and teratozoospermia. Obesity is associated with an inverse relationship between increases in gene/protein expression levels and declines in sperm quality. For instance, Shi et al. [30] found that high levels of protein-tyrosine phosphatase 1B (PTP1B) expression and activity were associated with the appearance of a defect in sperm acrosome reaction (AR) in sperm of obese mice. Zhao et al. [31] reported that testicular oxidative stress in mice on a HFD was related to declines in CAT and GSH-Px activity. On the other hand, decreased Crisp4 expression in testis and epididymis in the HFD group may be a cause of declines in reproductive success [32]. A previous study found that the methylation percentages at MEG3, SNRPN and SGCE/PEG10 differentially methylated regions (DMRs) significantly decreased in sperm of overweight or obese individuals. Furthermore, DNA methylation of DMRs increased on MEG3-IG and H19 in their sperm [33]. However, the obesity-induced mechanisms and key molecules are still ambiguous that contribute to poor sperm quality development.

To clarify how obesity reduces sperm quality, we addressed this question by using the described HFD model in mice [16, 34]. There is a general consensus that HFD-induced obesity is associated with declines in sperm motility and increases in sperm deformity [14, 35–37]. Meanwhile, there is also evidence showing impaired mitochondrial activity and increased sperm DNA damage caused by increases in ROS generation in sperm of the obese mice fed a HFD [38]. On the other hand, proteomic study of sperm is a more revealing approach to clarify the identity of key factors regulating sperm quality. This procedure is the most suitable because spermatozoa released from testis are transcriptionally and translationally suppressed and their functional maturation in epididymis is completely totally dependent on post-translational modifications [39]. The proteomic approach can identify differences in sperm protein profiles between normal and obese individuals. The human sperm proteome datasets in the public domain contain 1056 proteins including Triton-X soluble and insoluble fractions [40] and 1429 proteins in the dissociated head and tail fractions [41]. Mayank et al. [42] identified 667 different proteins from normozoospermic and asthenozoospermic sperm samples, and 5 proteins which were significantly down-regulated in asthenozoospermia containing diversiform nodes related to sperm motility, such as Ninein, Fascin-3 and Plexin-B2. Mahmoud et al. [43] compared the proteins in the sperm tail from normozoospermia and asthenozoospermia, and identified 4 novel proteins, i.e. HSPA9, TUBB2B, SPANX B and ASRGL1, which were also involved in asthenozoospermia. Our previous report describing the proteomic analysis of human obese athenozoospermic sperm showed that downregulation of endoplasmic reticulum protein 57 (ERp57) and actin-binding-related protein T2 (ACTRT2) correlates with declines in sperm quality [17].

In this study, the proteomics approach analyzed sperm protein expression patterns in obese mice fed a HFD. Out of 1562 proteins that were identified, the expression levels of 192 proteins were statistically significant different between the HFD and CD groups (*P* < 0.05). Some of these downregulated proteins in the HFD group are associated with an array of functions including cell structure and motility, endocytosis, transfer/carrier protein, actin and actin related protein, and cytoskeletal architecture. Given these associations, they may be relevant to spermiogenesis, a process that transforms the morphology of the unpolarized spermatids into a uniquely shaped spermatozoon. In mammals, this remodeling change includes acrosome biogenesis, head shaping, nuclear formation, flagellum formation and the removal of residual cytoplasm. In this processes, cytoskeletal structures, such as the acroplaxome and manchette, are necessary to support spermatid remodeling and sperm function [44, 45]. Meanwhile, endoplasmic reticulum proteins or vesicle trafficking-related proteins, such as GOPC [46], PICK1 [47], VPS54 [48], SMAP2 [49] and ATG7 [50], regulate proacrosomal vesicle transport from the Golgi to the acrosome and contribute to acrosome biogenesis and sperm head organization. Thus, the decreased expression of cytoskeletal proteins and vesicle proteins in sperm from obese mice might induce disturbed spermiogenesis, finally leading to disrupted and maladaptive sperm function. In our proteomic data, the cytoskeleton related proteins among the differentially expressed proteins in the HFD group, such as calicin [19], cylicin [19, 20], myosin [21], dynein [22, 23] and septin [24], are found to participate in spermiogenesis, maintaining sperm head shape and sperm motility. Moreover, there are still protein candidates in our proteomic data potentially valuable for further study on spermiogenesis and sperm function. For instance, secretory carrier-associated membrane proteins (SCAMP1 and SCAMP2), and VAMP-associated protein (VAPA) are involved in endomembrane dynamics and vesicle traffic [51–55], which can be conceivable candidates involved in maintaining spermiogenesis and male fertility.

Notably, we are aware of the limitation of LC-MS analysis. Such high throughput proteomics technologies only can be used as a biomarker discovery tool. Their putative identity requires validation before they can be used with confidence to resolve mechanisms underlying responses to environmental cues. Our proteomic data reveals that CSPP1 and CETN1 which are cytoskeletal proteins are the two candidates of the differentially expressed multifunctional proteins. We paid particular attention to these target proteins because one of them is CETN1, a well-characterized calmodulin-like Ca^2+^ binding protein expressed in all eukaryotic ciliated cells from yeast to mammals. It is expressed in the photoreceptor cells and other ciliated cells in rodents, including sperm [56]. It was shown that *Cetn1* knock out male mice were sterile, which is associated with abnormal head morphology and reduced or absence of middle and main tail segments, indicating a crucial role for this protein in spermiogenesis [25]. Herein, this is the first report describing a relationship between CETN1 expression levels and obesity-associated asthenozoospermia and teratozoospermia.

CSPP1 is a cytoskeletal protein related to centrosome/microtubule cytoskeleton and spindle formation [26]. Some reports documented that a CSPP1mutation is the main cause of Joubert syndrome (JBTS), a type of invisible cilia and Jeune asphyxiating thoracic dystrophy (JATD) [27], whereas overexpression of CSPP1 in hTERT-RPE cells can result in longer cilia [57]. The loss of human CSPP1 function may affect the formation and length of primary cilia, and axonal transport of ciliary proteins, but no studies reported that it was relevant to male fertility or sperm function. Our data showed that CSPP1 is highly expressed in testis and enriched in the post-acrosomal half of the spermatids, which are situated parallel to the microtubule tracks of the manchette. To further delineate this alleged relationship between CSPP1 and obesity induced poor sperm quality, clinical semen parameters were evaluated and the results confirmed that overweight and obesity are both associated with asthenozoospermia and teratozoospermia. Furthermore, Western blot analysis verified that low CSPP1 expression accompanies obesity-associated human astheno-teratozoospermia. Additionally, CSPP1 localization in the sperm head–tail coupling apparatus also suggests that this protein may take part in sperm head shaping or flagellum formation during spermiogenesis. Therefore, reduced expression of CSPP1 in obese testis and sperm may contribute to disrupted and maladaptive cytoskeletal structure and sperm deformity. Whereas additional studies are required to understand precisely how CSPP1 expression in the spermatids involves in sperm head shaping and how obesity leads to declines in CSPP1 expression, our immediate goal was to set the stage for assessing the correlation of CSPP1with obesity associated asthenozoospermia and teratozoospermia.

## Conclusions

In the HFD induced obese mice model, differential proteomic analysis identified a potential mechanism wherein changes in the CSPP1 and CETN1 cytoskeletal protein expression levels alter spermatid remodeling during spermiogenesis and underlie declines in sperm quality. Moreover, we demonstrated that CSPP1 and CETN1 is expressed in spermatocytes and spermatids in mouse testis and its distribution is related to the manchette structure that is crucial to spermatid remodeling and sperm function. Meanwhile, low CSPP1 and CETN1 expression levels are associated with human astheno-teratozoospermia in clinical samples. Taken together, these data suggest that regionally delimited expressions of CSPP1 and CETN1 are strongly associated with spermiogenesis and maintenance of normal sperm morphology whereas its deficiency in sperm may contribute to obesity-associated asthenozoospermia and teratozoospermia. These newly identified candidates may become useful functional markers for further unraveling how obesity leads to declines in sperm quality and male fertility.

## Supplementary information

**Additional file 1: Table S1.** Proteins identified in the sperm proteome.

## Data Availability

All data generated or analyzed during this study are included in this published article and its supplementary information files.

## Abbreviations

AR: Acrosome reaction
BSA: Bovine serum albumin
CASA: Computer-assisted sperm analysis
CD: Control diet
CETN1: Centrin-1
CSPP1: Cytoskeletal proteins centrosome and spindle pole associated protein 1
FATs: Functional annotation terms
GO: Gene ontology
H&E: Hematoxylin and eosin
HFD: High-fat diet
IF: Immunofluorescence
IHC: Immunohistochemistry
JATD: Jeune asphyxiating thoracic dystrophy
JBTS: Joubert syndrome
KEGG: Kyoto encyclopedia of genes and genomes
KO: Knock-out
LC-MS: Liquid chromatography tandem mass spectrometry
LC-MS/MS: Liquid chromatography-tandem mass chromatography
MMAF: Multiple morphological abnormalities of the sperm flagella
PVDF: Polyvinylidene difluoride
SPSS: Statistic package for social science
STD: Standard deviation; T test: student’s t-test
